# Remarks on Cholera

**Published:** 1824-10

**Authors:** B. L. Sardham

**Affiliations:** Surgeon to the Forces.


ART. VI.—*Remarks on Cholera.* Being an Extract of a Letter from B. L. SARDHAM, Esq. Surgeon to the Forces.

WE have had the most distressingly hot and oppressive season I ever recollect, with very little rain, and that not till late in the season, which was, of course, productive of disease in its most acute form; fevers, with great determination to the head and liver, calling for the most prompt and decisive treatment, chiefly by the lancet, with active purging, &c. They were cases which kept me constantly on the alert, and admitted of little rest; but the result has amply repaid me.

I have been paying the closest attention to cholera, and have kept the few cases I have lately had, most minutely,—indeed, night and day; but, whether I shall ever have it in my power to generalize on the subject to any good purpose, time and opportunity must decide. What renders it so very difficult is, its having, even within my own observation, undergone such modifications, and assumed such varied shapes; and, from what I have casually heard, still greater varieties have occurred in other parts of the country. Nine well-marked cases presented themselves in May and June, the two first of which terminated fatally, and the morbid appearances in them have produced a very considerable change in my ideas as to the nature of the disease; and, I believe, enabled me to save the rest. I have been impressed with the opinion that it is in consequence of irregular or suspended action of the brain, all the other symptoms arise: the circulation is almost stopped; the blood retires from the surface; the heart is incapable of its office; congestion takes place in the head, and particularly the liver; the functions of the kidneys are suspended; violent cramps of the limbs, indeed of the whole body, take place, generally commencing in the toes and fingers, running often to the trunk; the abdominal muscles frequently drawn up into hard knots, or balls, as large as a turkey's egg; the inner coats of the stomach puckered up into deep folds, and the stomach itself almost into two separate bags; the calibre of the small intestines, in different parts, reduced or contracted so much as to be almost impervious, and their villous coat dry, and lined with a viscid mucus, feeling between the fingers like paste; the bladder a small and (as it were) solid ball, with a cavity which would not contain a nutmeg: indeed, all the secretions are suspended, save the diseased watery one from the stomach and lower intestines.

When I first had to encounter this disease, I objected to, and did not use, the lancet, because I knew inflammation of the stomach and intestines to be a consequence, and not a cause; for, in all those in whom it was most acute and terminated fatally in a few hours, neither the stomach nor intestines ever showed a red vessel, but there was the greatest venous congestion. But, since the change in my ideas relative to the nature of the disease, I have, where I could get it, immediately bled to the extent of from forty to even sixty ounces, and with the most marked good effects; the patients finding great and immediate relief, the heart resuming its office, the pulse returning to the wrist, the surface losing its shrunken livid appearance and deadly coldness, and the cramps, vomiting, and peculiarly distressing and implacable thirst, being greatly moderated. The bleeding has been immediately followed by the exhibition of from one to two ounces of castor-oil, with from half to an ounce of oleum terebinthinæ, which generally almost immediately checks the vomiting, and removes the spasm; and, from its antispasmodic and purgative action, large watery, muddy, greenish, or black evacuations, are procured. If vomiting and spasm are not checked by the first dose, it is repeated in one, two, or three hours, according to the urgency of the case, with a blister over the stomach. I have then given a full dose of calomel, followed, a few hours after, by a couple of drachms of compound powder of jalap, which generally brings away a load of green or black bilious matter, to the almost certain relief of the sufferer. The jalap is not given where any disposition to spasm remains, but a couple of ounces of castor-oil; and should it have again been excited by the jalap, which it not unfrequently is, the oil is almost certain to relieve it, but particularly when combined with the turpentine. The calomel appears to me to be perfectly useless, if given before the oil and turpentine, which quickly and certainly finds a passage, and seems to remove the spasm of the stomach and constriction of the small intestines. Indeed, unless the calomel is preceded by it, I believe it seldom passes the pylorus; for, at the time it was cried up as the sovereign remedy in 1817, I saw it constantly rejected; and, when it was not, I found it in the stomach.

I am now, of course, speaking of the disease in Europeans; for opium, in large doses, with natives often succeeded, although in the former I never saw it do any good: indeed, any strong spirit often did relieve the natives.

The warm-bath I have given a fair trial to, but, in cases of pure cholera, I have never seen more than temporary relief from it while in the water, and most of the worst symptoms appeared aggravated by it on coming out: indeed, it seemed to increase mischief in many, and the cries of some were redoubled on immersion,—they could not bear it. How is this to be accounted for? I think by its increasing the congestion in the head, and thus still more oppressing the brain, the powers of which are already, as I conceive, diminished, if not suspended. I deeply regret that my dissections in these cases, in 1817, when I had such ample opportunity of seeing it in its most fatal shape, did not extend to the head; yet such was the pressure of the moment, and being for the greatest part of the time alone, I really had not leisure: the appearances, however, found in the crania of the two I lost this season, now account to me for the state in which I saw some die in 1817.

In October and November, bilious remittent fever made its appearance in quite an epidemic form, but, although acute in its attack, it was ductile to treatment, and generally suddenly yielded. I did not lose a patient: very few escaped it, officers, women, or children, and patients in hospital. In November, violent spasms or cramps frequently succeeded the attack, always attended with giddiness, within three or four hours, precisely of the same appearance as in cholera, save that the skin was hot, with an oppressed hard pulse; but in these the warm bath was decidedly beneficial, to which sometimes a full bleeding, with the oil and turpentine, they almost immediately yielded.

*Meerat; December,* 1823.

